# Phosphorus-nitrogen compounds- (Part 50): correlations between structural parameters for cylophosphazene derivatives containing ferrocenyl pendant arm(s)

**DOI:** 10.3906/kim-1909-83

**Published:** 2020-06-01

**Authors:** Nuran ASMAFİLİZ, Gamze ELMAS, Aytuğ OKUMUŞ, Selen BİLGE KOÇAK, Zeynel KILIÇ

**Affiliations:** 1 Department of Chemistry, Faculty of Science, Ankara University, Ankara Turkey

**Keywords:** **spiro** cyclic Ferrocenyl phosphazene, ^31^P NMR, X-ray crystallography, endocyclic bond angle, exocyclic bond angle, electron density transfer parameter

## Abstract

The results of a systematic study of **spiro** -cyclotri/tetraphosphazenes with ferrocenyl pendant arm on the basis of correlation between structural parameters were presented. The main parameters were obtained from Xray crystallography and ^31^P NMR results in order to investigate the relationship between the δ P_**spiro**_ shift values and endocyclic and exocyclic NPN bond angles, and electron density transfer parameters. Structural parameters derived from 11 types of the ferrocenyl cyclophosphazene derivatives with 5- to 7-membered **spiro** -rings introduced to the literature from our research group were studied and compared with each other.

## 1. Introduction

The phosphazene chemistry has attracted much attention since 1960 [1,2]. Especially, hexachlorocyclotriphosphazene (N_3_P_3_Cl_6_, trimer) and octachlorocyclotetraphosphazene (N_4_P_4_Cl_8_, tetramer) are of particular interest to both theoretical and experimental researchers concerning phosphazene-based chemistry. Because of their tendency to react with the nucleophilic mono-, di-, or multi-functional groups [3–6], both of the cyclophosphazenes were used in the syntheses of a considerable range of organocyclotri/tetraphosphazene derivatives with diverse applications [7,8]. The substantial efforts have been performed on the nucleophilic substitution reactions, in which the 1- to 6-Cl-atoms on trimer and 1- to 8-Cl atoms on tetramer have been replaced by the NH and/or OH functioned reagents, forming isomeric products e.g., structural ( *spiro* -, *ansa* - and *bino* -architectures or a mixed of the same or different architectures), geometrical (geminal, non-geminal *cis/trans* -), and optical isomers [9,10]. The nature of the products strongly depends on the various chemical factors which control the replacement reaction mechanisms such as chain lengths of nucleophilic groups, the polarity of solvents, and the reaction temperature [11]. So far only a limited number of published studies on cyclophosphazene derivatives with ferrocenyl pendant arm is present in the literature [12–16].

The organocyclophosphazene derivatives have several potential applications in different fields of science as flame-retardant additives for organic polymers [17], liquid crystals [18,19], antibacterial [20] and anti-cancer [21] agents, fluorescence chemosensors [22], ion-transferring agents for rechargeable lithium batteries [23,24] and light-emitting diodes (LEDs) [25]. Besides, ferrocene-containing compounds have been used for molecular sensors, biosensors, electron-transfer mediators, non-linear optical materials, liquid crystals, and redox-active probe materials [26,27]. In this context, we were therefore interested in synthesizing of ferrocenyl cyclophosphazenes and thought that the presence of both ferrocene moiety as a substituent and a trimeric/tetrameric phosphazene as a skeleton in a molecule could give rise to a novel kind of cyclophosphazene derivatives and bring together many biological and physicochemical properties of the molecule. Furthermore, cyclotri/tetraphosphazene ring systems are electrochemically inert, and ferrocenyl group is an excellent redox-active precursor. Hence, ferrocenyl cyclophosphazenes were synthesized to investigate the electrochemical behavior of the phosphazenes [28–30]. Furthermore, substituted *spiro* -monoferrocenyl cyclotri/tetraphosphazenes were prepared by our group to evaluate in terms of their antituberculosis, anticancer, and antimicrobial activities. According to these studies, it was observed that geminal vanillinato (Van)-substituted *spiro* -monoferrocenyl cyclotriphosphazenes [31], tetra pyrrolidine (Pyr)-substituted *spiro* -mono [32,33] and bisferrocenyl [33] cyclotriphosphazenes and hexa Pyr-substituted *spiro* -monoferrocenyl cyclotetraphosphazenes [34] inhibited the growth of *Mycobacterium tuberculosis* H37Rv. While 1,4-dioxa-8-aza *spiro* [4,5]decane (DASD)-substituted *spiro* -bisferrocenyl [35] and partly substituted di *spiro* -bisferrocenyl [36] cyclotriphosphazenes, the fully and nongeminal (cis) [37] Van-substituted *spiro* -monoferrocenyl cyclotriphosphazenes, were effective against the human cervical cancer cell line (HeLa), bis(diamino) substituted di *spiro* -bisferrocenyl cyclotetraphosphazene was found to be more active against colon cancer DLD-1 cells than *Doxorubicin* [38]. It was also found that the DASD and Pyr-substituted ferrocenyl cyclotriphosphazenes were active against some gram-positive and gram-negative bacteria [32,33,35] and Pyrsubstituted ferrocenyl cyclotetraphosphazenes were more effective than the commercial antifungal drug *Ketoconazole* against fungi [34].

Besides, the chiral properties of mono Van-substituted di *spiro* -bis ferrocenyl cyclophosphazenes were investigated by ^31^P NMR spectroscopy upon the addition of the chiral solvating agent [39].

On the other hand, we also succeeded in the preparation of ultrathin and highly ordered Langmuir-Blodgett films of tetrachloro-, and mono and gem DASD-substituted mono-ferrocenyl cyclotriphosphazenes [40,41]. These compounds are the first phosphazene derivatives prepared as thin films in the literature.

Shaw described the first systematic study of the relationship between the bond angles around the phosphorus atoms and ^31^P NMR spectral data in phosphazene derivatives [42]. The changes in structural parameters for different kinds of structurally analogous cyclotriphosphazenes (cyclotriphosphazenes possessing 6-membered *spiro* ring/rings [43], mono *spiro* -, di *spiro* -, *spiro* - *ansa* - *spiro* - and *spiro* - *bino* - *spiro* -cyclotriphosphazenes [44–46], *spiro* -cyclotriphosphazenic lariat (PNP-pivot) ether derivatives [47,48], monotopic and ditopic *spiro* -crypta cyclotriphosphazenes [49–51]) were investigated previously. It was found that the systematic variations in the ^31^P NMR chemical shifts depend fundamentally on some electronic (electron-releasing and electron-withdrawing capacities of substituent groups), steric (the steric hindrance between the exocyclic groups) and conformational factors, and on the changes in bond lengths and bond angles around the phosphorus atoms [especially endocyclic (α) and exocyclic (α′) bond angles] in cyclotriphosphazene derivatives. The current study deals with a number of correlations between structural parameters [e.g., ^31^P NMR spectral data and X-ray crystallographic data (endocyclic and exocyclic NPN bond angles, and bond lengths)] in *spiro* cyclic ferrocenyl cyclophosphazenes introduced to the literature from our research group (Table 1) [33–41,52]. Therefore the content of this report includes: (i) a brief description of the synthesis methods of 11 different structural types and a total of 28 *spiro* cyclic ferrocenyl phosphazenes with 5- to 7−membered *spiro* -rings used for the graph construction, (ii) the relationship between the δ P_*spiro*_ shifts and the values of electron density transfer parameters Δ(P–N), and (iii) the correlation of δ P_*spiro*_ shifts and endocyclic (α) and exocyclic (α′) NPN bond angles of the compounds.

**Table 1:** The endocyclic (α) and exocyclic (α′) NPN bond angles and bond lengths (a, a′ , b, and b′) on the formulae of cyclophosphazenes.

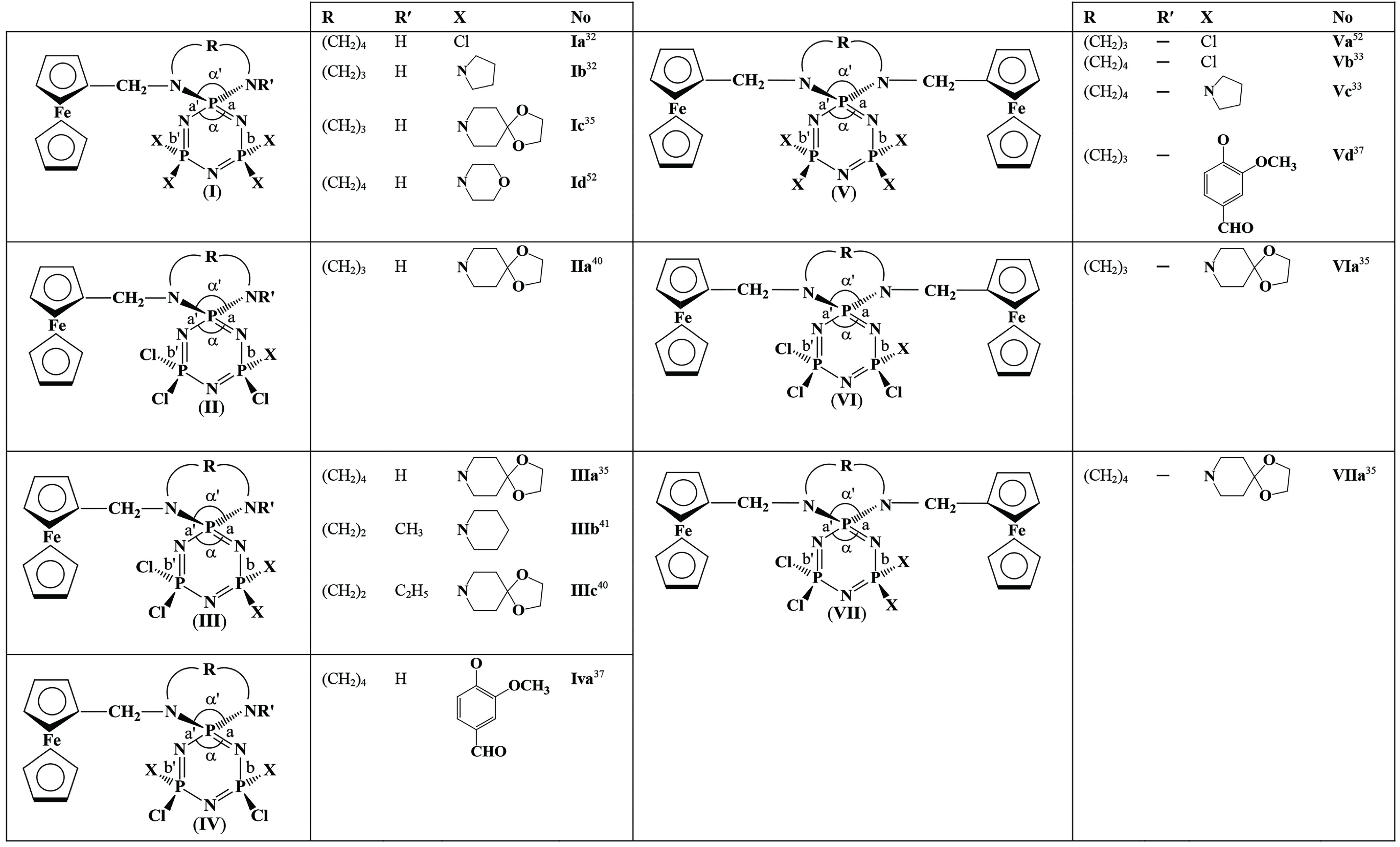
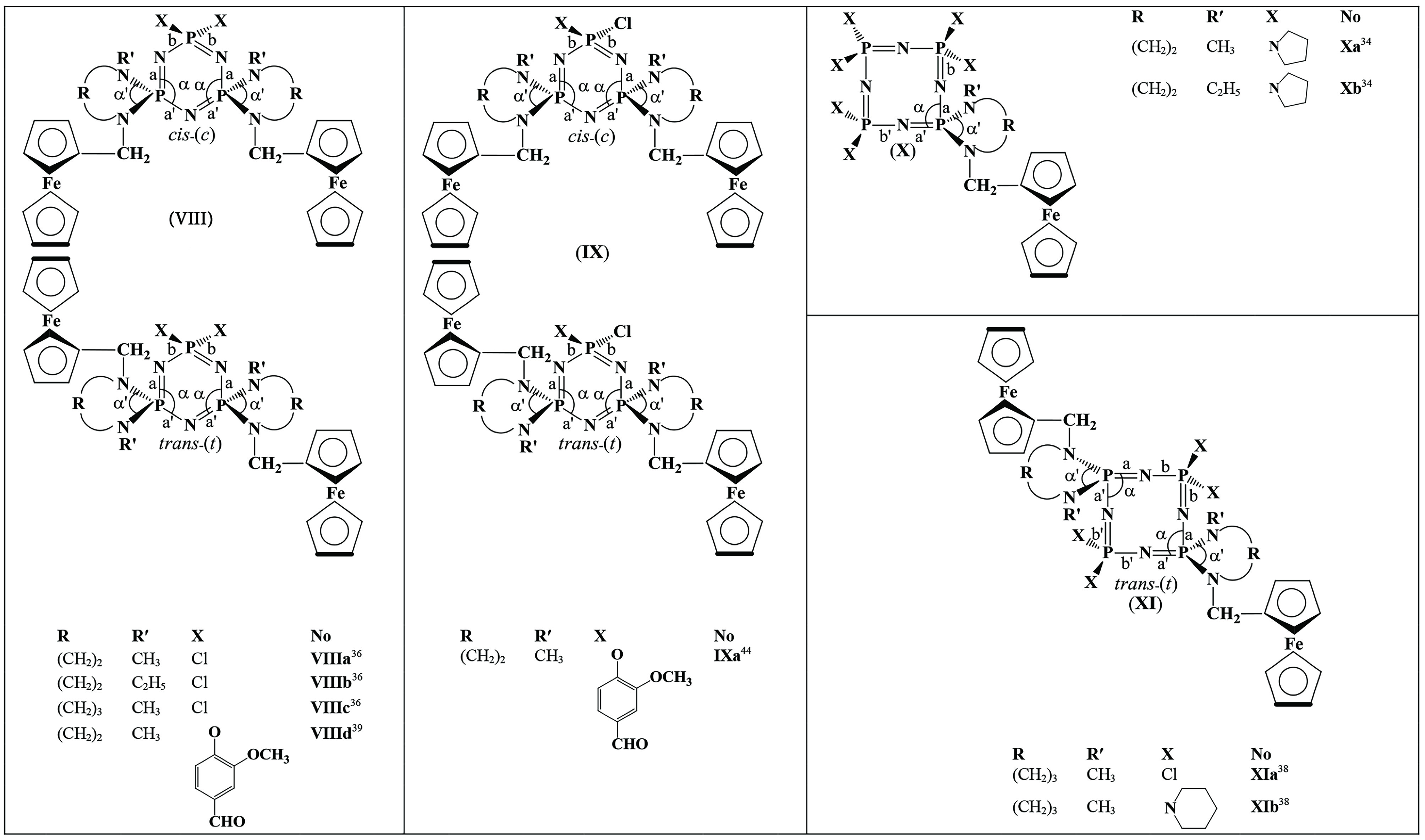

## 2. Results and discussion

### 2.1. Syntheses

Routes for the synthesis of *spiro* cyclic ferrocenyl cyclophosphazenes clarified their solid-state structures using X-ray crystallography by our research group and investigated in this study are summarized in Scheme. The syntheses of mono and bisferrocenyl diamines, as the starting compounds, were carried out according to the published procedures, in which ferrocenecarboxaldehyde reacted with appropriate diamines and followed by reduction of the azomethine bonds in the intermediate products [53,54]. The reactions of trimer with mono and bisferrocenyl diamines gave partly substituted *spiro* -mono ( **I** ) [33] and *spiro* -bis ( **V** ) [33,52] ferrocenyl cyclotriphosphazenes, respectively. The substituted phosphazene derivatives were synthesized by stepwise substitutions of partly substituted *spiro* -mono ( **I** ) and *spiro* -bis ( **V** ) ferrocenyl cyclotriphosphazenes which consist of 4 reactive P-Cl units. The reactions of 1 equimolar amount of partly substituted *spiro* -bis ( **V** ) and *spiro* mono ( **I** ) ferrocenyl cyclotriphosphazenes with 1 and 2 equimolar amounts of heterocyclic amines (DASD and Pyr) produced corresponding mono heterocyclic amine (DASD) substituted *spiro* -bis (VI) [35] and *spiro* -mono ( **II** ) [40] and geminal heterocyclic amine (DASD and Pyr) substituted *spiro* -bis ( **VII** ) [35] and *spiro* -mono ( **III** ) [35,40,41] ferrocenyl cyclotriphosphazenes in the presence of NEt3 in refluxing dry THF. The fully heterocyclic amine [DASD, Pyr, and morpholine (Morp)] substituted *spiro* -bis ( **V** ) [33] and *spiro* -mono ( **I** ) [33,35,52] ferrocenyl cyclotriphosphazenes were prepared by replacing 4 Cl-atoms on partly substituted derivatives ( **I** ) and ( **V** ), respectively, with excess heterocyclic amines in boiling THF. The reactions of equimolar amounts of partly substituted *spiro* -mono ferrocenyl cyclotriphosphaze ( **I** ) and potassium vanillinate were found to yield the corresponding mono Van-substituted *spiro* -mono ferrocenyl cyclotriphosphaze ( **III** ) as a major product and geminal ( **III** ) [37] and nongeminal (cis) ( **IV** ) substituted *spiro* -mono ferrocenyl cyclotriphosphazenes as minor products. Fully Van-substituted *spiro* -bisferrocenyl cyclotriphosphazene ( **V** ) was synthesized from the reaction carried out with excess potassium vanillinate [37]. The Cl-replacement reactions of trimer with 2 equimolar amounts of mono-ferrocenyldiamines resulted in the formation of the corresponding partly substituted cis- (meso) and trans-(racem) di *spiro* -bisferrocenyl cyclotriphosphazenes ( **VIII** ) as the major products and *spiro* -mono ( **I** ) ferrocenyl cyclotriphosphazenes as minor products [36]. Three products were separated performing column chromatography. The reactions of 1 equimolar amount of cis- and trans-di *spiro* -bisferrocenyl cyclotriphosphazenes ( **VIII** ) having 2 reactive Cl-atoms with 2 equimolar amounts of potassium vanillinate in refluxing THF afforded the mono ( **IX** ) and fully ( **VIII** ) Van-substituted cis- and trans-di *spiro* -bisferrocenyl cyclotriphosphazenes ( **IX** ) and ( **VIII** ) [39]. The mono and fully substituted derivatives were separated using column chromatography. On the other hand, the partly substituted *spiro* -mono ( **X** ) [34] and cis- and trans-di *spiro* -bis ( **XI** ) [38] ferrocenyl cyclotetraphosphazenes were obtained from the reactions of tetramer with 1 and 2 equimolar amounts of monoferrocenyl diamines in dry THF. The fully Pyr-substituted ( **X** ) and trans-( **XI** ) were prepared by the reaction of partly substituted ones with excess Pyr in dry THF at ambient temperature.

**Scheme 1:**
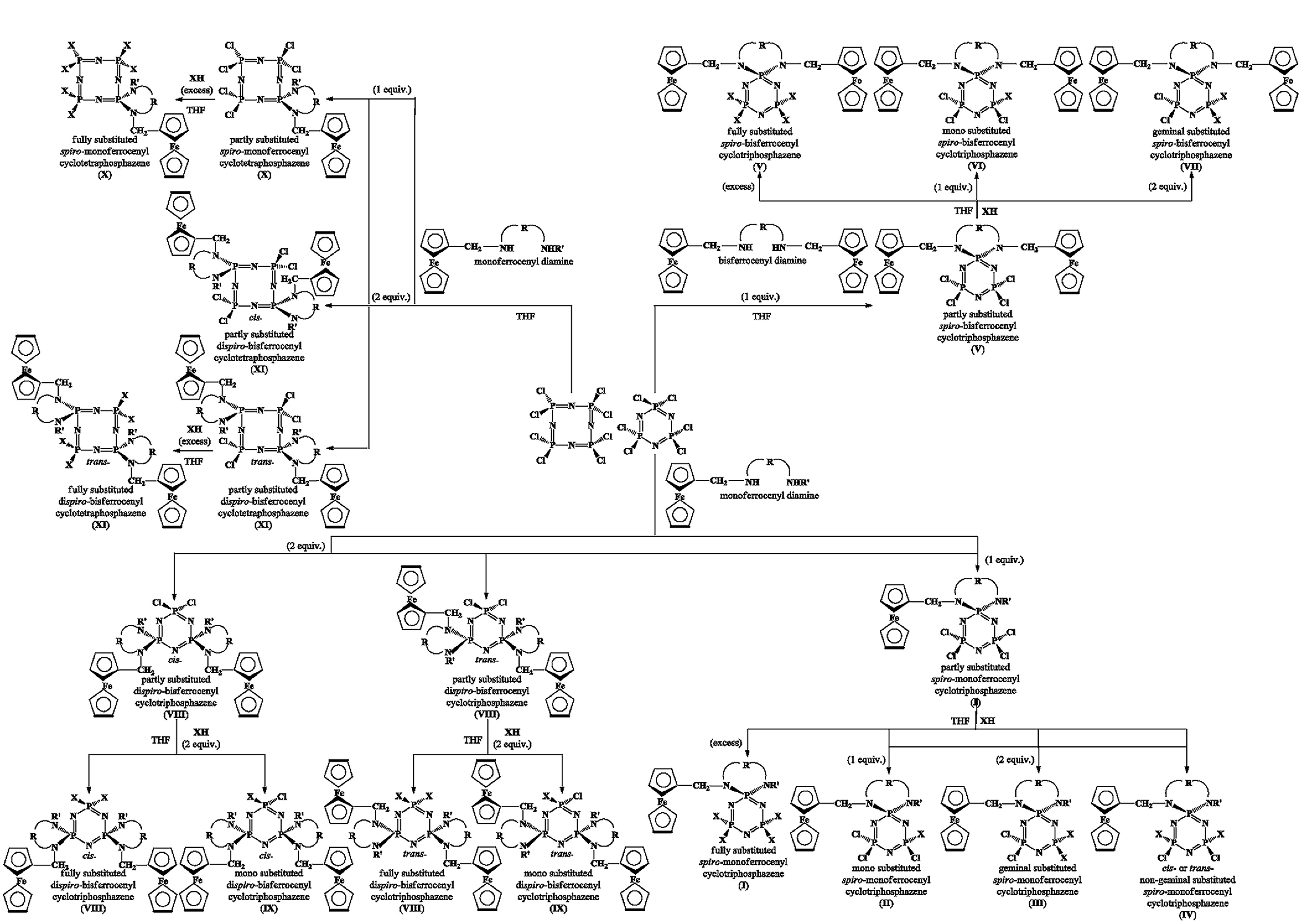
Routes for the synthesis of spirocyclic ferrocenyl cyclophosphazene derivatives investigated in this study.

### 2.2. Correlations between structural parameters

The endocyclic (α) and exocyclic (α′) NPN bond angles, and the bond lengths (a, a′ , b, and b′) were defined in the generalized structures for the 11 types of cyclotri/tetraphosphazenes containing ferrocenyl pendant arm/arms and 5-, 6- and 7-membered *spiro* -ring/rings shown in Table 1. δP_s_ *piro* shifts, α, and α′ bond angles, and Δ(P–N) values that are needed to be used for graph construction are listed in Table 2. The corresponding values of the standard compounds trimer (N3 P3 Cl6) [55,56] and tetramer (N4 P4 Cl8) [57,58] were taken from the literature. Types **I** and **V** members are partly and fully substituted *spiro* -mono and *spiro* -bisferocenyl cyclotriphosphazenes, respectively. Mono and geminal substituted *spiro* -mono/bisferocenyl cyclotriphosphazenes are the types **II** and **VI** , and the types **III** and **VII** group members, respectively. Nongeminal (cis) substituted *spiro* -monoferocenyl cyclotriphosphazene constitutes the type **IV** . Members of types **VIII** and **IX** derivatives comprise partly and fully substituted and monosubstituted *cis/trans* -di *spiro* -bisferocenyl cyclotriphosphazenes, respectively. *spiro* -Mono and *trans* -di *spiro* -bisferocenyl cyclotetrahosphazenes constitute the types **X** and **XI** compounds.

**Table 2:** Endocyclic (α) and exocyclic (α′) NPN bond angles, bond lengths (a, a′, b, and b′), δP_*spiro*_ shifts and Δ(P-N) values for the compounds [δP_*spiro*_shifts in ppm, α and α′ angles in °, a, a′, b, and b′ lengths in Å].

Compound	a	a'	b	b'	Δ(P-N)	α	α'	ΔP_NPN_	
**Ia** ^33^	1.613(3)	1.614(3)	1.548(3)	1.548(3)	0.0870	113.14(15)	102.68(16)	13.62	for ( **I-VII** ), ( **X** ) and ( **XI** ) Δ (P-N) = (a+a')/2-(b+b')/2 for ( **VIII** ) and ( **IX** ) Δ (P-N) = (a+a')/2 - b
**Ib** ^33^	1.587(3)	1.598(3)	1.603(3)	1.588(2)	-0.003	117.66(13)	102.16(13)	20.76	
	1.589(2)	1.600(3)	1.601(3)	1.587(3)	0.0005	117.73(13)	101.86(13)		
**Ic** ^35^	1.586(1)	1.600(1)	1.602(1)	1.594(1)	-0.005	117.67(17)	103.02(7)	19.38	
**Id** ^52^	1.604(1)	1.597(1)	1.593(1)	1.597(1)	0.0055	115.47(7)	104.06(7)	22.10	
**IIa** ^40^	1.630(8)	1.596(8)	1.547(9)	1.566(8)	0.0565	113.7(4)	101.7(4)	17.00	
**IIIa** ^35^	1.592(1)	1.628(1)	1.592(1)	1.560(1)	0.034	114.62(6)	104.68(6)	17.32	
** IIIb** ^41^	1.579(3)	1.616(3)	1.598(3)	1.562(3)	0.0175	113.18(15)	93.24(15)	22.06	
**IIIc** ^40^	1.6208(10)	1.5903(10)	1.5634(10)	1.5921(10)	0.0278	113.32(5)	95.00(5)	21.06	
**IVa** ^37^	1.612(2)	1.612(2)	1.555(2)	1.556(2)	0.0565	113.50(10)	100.13(10)	15.41	
**Va** ^52^	1.620(3)	1.620(3)	1.544(3)	1.544(3)	0.076	110.0(2)	105.0(2)	6.20	
**Vb** ^33^	1.613(3)	1.619(3)	1.549(3)	1.548(3)	0.0675	113.09(13)	103.14(13)	14.56	
**Vc** ^33^	1.599(4)	1.591(3)	1.592(3)	1.591(4)	0.0035	114.96(18)	98.94(17)	22.08	
**Vd** ^37^	1.613(3)	1.613(3)	1.563(3)	1.563(3)	0.05	113.3(2)	100.6(2)	21.60	
**VIa** ^35^	1.592(2)	1.626(2)	1.574(2)	1.554(2)	0.045	114.42(13)	102.85(11)	14.41	
**VIIa** ^35^	1.558(2)	1.600(2)	1.603(3)	1.593(2)	-0.019	113.26(9)	101.45(9)	18.32	
***t* -VIIIb** ^36^	1.620(4)	1.587(3)	1.555(4)	-	0.0485	113.31(17)	94.49(18)	24.22	
	1.610(3)	1.587(3)	1.561(4)	-	0.0375	113.05(17)	94.48(17)		
***c* -VIIIb** ^36^	1.618(3)	1.593(3)	1.552(3)	-	0.0535	112.98(16)	94.33(18)	22.57	
	1.620(3)	1.586(3)	1.565(3)	-	0.038	113.25(16)	93.94(18)		
***t* -VIIIb** ^36^	1.614(3)	1.589(3)	1.558(3)	-	0.0435	113.24(16)	94.61(16)	22.63	
	1.612(3)	1.589(3)	1.569(3)	-	0.0315	112.23(16)	93.92(16)		
***c* -VIIIc** ^36^	1.634(2)	1.583(2)	1.549(3)	-	0.0595	113.84(13)	102.10(13)	19.63	
	1.610(2)	1.594(2)	1.572(3)	-	0.03	115.59(12)	103.91(14)		
***t* -VIIIc** ^36^	1.6055(19)	1.5951(17)	1.5602(19)	-	0.0401	115.57(9)	104.35(10)	19.65	
	1.6292(19)	1.5837(17)	1.5585(19)	-	0.04795	114.08(9)	103.63(9)		
***c* -VIIId** ^39^	1.6014(19)	1.588(2)	1.5716(18)	-	0.0231	114.03(10)	95.22(9)	27.32	
	1.601(2)	1.5913(19)	1.5647(19)	-	0.03145	113.42(10)	93.39(10)		
***t* -VIIId** ^39^	1.608(3)	1.592(4)	1.566(3)	-	0.034	113.43(17)	93.75(19)	27.52	
	1.611(3)	1.586(3)	1.573(3)	-	0.0255	113.56(18)	94.72(18)		
***c* -IXa** ^39^	1.605(2)	1.5922(19)	1.562(2)	-	0.0366	113.64(10)	94.54(10)	26.68		
	1.612(2)	1.586(2)	1.563(2)	-	0.036	112.50(10)	93.93(11)		
***t* -IXa** ^39^	1.610(3)	1.593(3)	1.560(4)	-	0.0415	113.77(19)	94.88(18)	25.91	
	1.622(3)	1.584(3)	1.550(4)	-	0.053	113.34(18)	93.89(19)		
**Xa** ^34^	1.587(3)	1.583(3)	1.589(3)	1.586(3)	-0.0025	118.19(15)	94.31(13)	12.61	
**Xb** ^34^	1.584(2)	1.581(2)	1.592(2)	1.588(2)	-0.0075	117.68(9)	94.94(8)	12.27	
**XIa** ^38^	1.5940(13)	1.5853(14)	1.5301(13)	1.5570(13)	0.046	114.91(7)	102.01(7)	1.19	
**XIb** ^38^	1.584(3)	1.573(3)	1.585(3)	1.582(3)	-0.005	121.07(14)	102.14(13)	3.46	
	1.582(3)	1.568(3)	1.584(3)	1.577(3)	-0.0055	119.87(14)	102.94(14)		

The concept of the double-bond character of the P-N linkage in the cyclophosphazene derivatives is still not clearly understood. Negative hyperconjugation and ionic bonding alternatives are exclusive [59]. The natural bond orbital and topological electron-density analyses of phosphazenes have proved the crucial role of negative hyperconjugation in the description of the P-N bond.

#### 2.2.1. The correlation of δ P_*spiro*_ shifts and values of electron density transfer parameters Δ(P–N)

**Figure 1:**
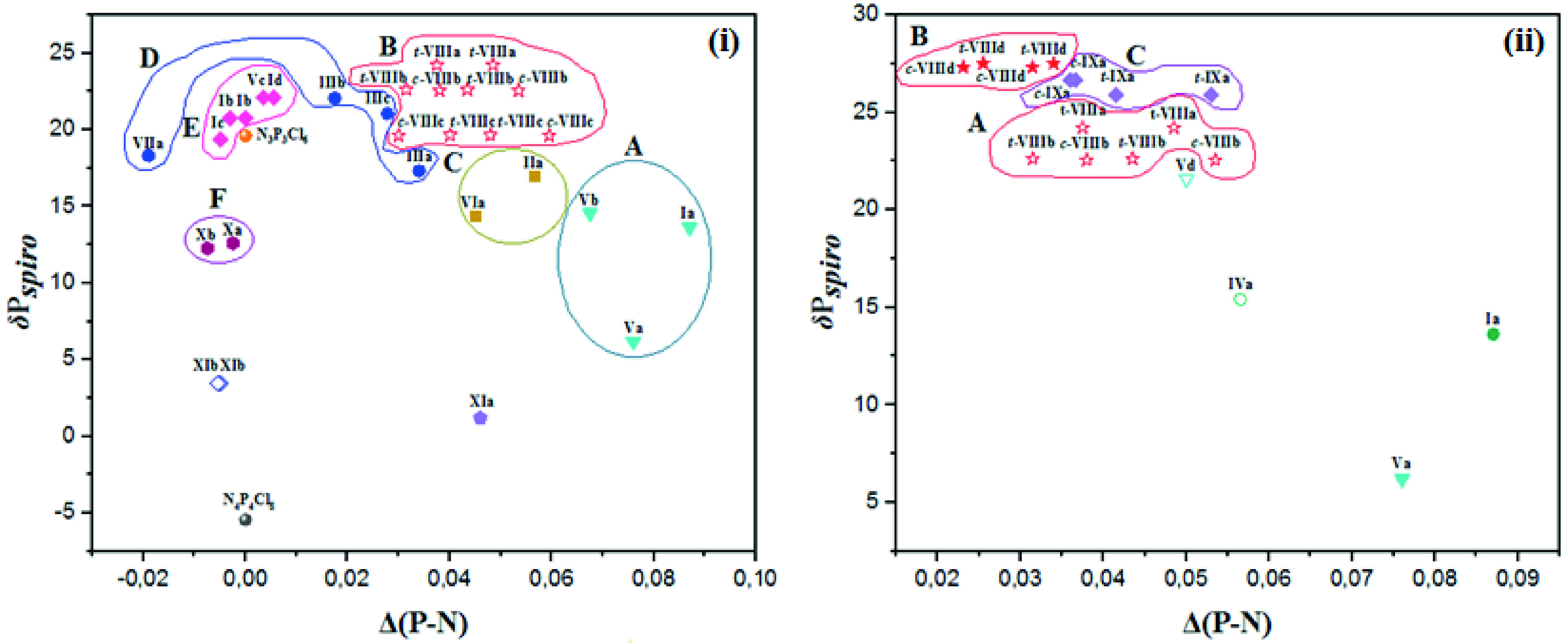
The relationship between δ P_*spiro*_ shifts and Δ(P-N) values for partly and heterocyclic amine (Pyr, Pip, Morp, DASD) (i) and Van (ii) substituted *spiro* cyclic ferrocenyl phosphazenes. δ P_ClPCl_ shift values of N_3_P_3_Cl_6_ and N_4_P_4_Cl_8_ are 19.60 [56] and –5.45 [58] ppm, respectively.

The electron density transfer parameter Δ(P–N) is the difference between the bond lengths of 2 adjacent endocyclic P-N bonds as defined in Table 2 for *spiro* cyclic ferrocenyl phosphazenes. It shows a measure of the electron releasing and withdrawing capacities of the substituent groups on cyclophosphazene ring. The relationship between the δ P_*spiro*_ shifts and Δ(P–N) values is illustrated in Figure 1 for partly and heterocyclic amine [Pyr, piperidine (Pip), Morp and DASD) (i) and Van (ii) substituted *spiro* cyclic ferrocenyl phosphazenes, respectively. A cluster of points rather than the linear trend was observed between the Δ(P–N) and δ P_*spiro*_ shifts. In Figure 1i, all types of triphosphazene structures were accumulated in 6 regions A, B, C, D, E, and F. The points of partly substituted types ( **I** and **V** ) and type **VIII** phosphazenes accumulate in regions A and B, respectively. The points of mono (types **II** and **VI** ), geminal (types **III** and **VII** ) and fully heterocyclic amine substituted cyclotri (types **I** and **V** ) and cyclotetra (type X) phosphazenes accumulate in regions C, D, E, and F, respectively.

According to Figure 1i, some comparisons can be made on the electron-releasing power of the substituent depending on whether the substituent is a chloro or heterocyclic amine group of the compounds with the same membered *spiro* -rings. For example, the Δ(P–N) values of fully heterocyclic amine substituted **I** d, **V** c, and ***t* -XI** b are respectively; 0.0055, 0.0035, and –0.005 and –0.0055, indicating that the electron releasing power of the nitrogen atoms of heterocyclic amine groups is greater than that of the chloro groups in Ia (0.087), **V** b (0.0675), and ***t* -XI** a (0.046) with the larger Δ(P–N) values. A similar situation is observed for fully Van ( **V** d and ***c/t* -VIII** d) and partly [ **V** a and ( ***t* -VIII** a and ***c/t* -VIII** b)] substituted ferrocenyl cyclophosphazenes (Figure 1ii), showing the oxygen atoms of Van groups bonded to phosphorus atoms release electrons to the cyclophosphazene ring. It is not possible to say whether heterocyclic amines or vanniline release more electrons to the phosphazene ring since we do not have crystallographic data of the heterocyclic amines and Van substituted derivatives of any type are not available.

Moreover, there is no significant difference between the Δ(P–N) values of *cis-* and *trans* -structures of the same compound for types **VIII** and **IX** phosphazenes (0.00825 for **VIII** b, 0.002475 for **VIII** c, and 0.01095 for **IX** a). However, the difference between the Δ(P–N) values of *cis-* and *trans* -structures of the phosphazenes with 5-membered *spiro* -rings ( **VIII** b and **IX** a) is slightly larger than that of the phosphazene with 6-membered *spiro* -rings ( **VIII** c). That could be significantly attributed to the fact that 5-membered *spiro* -rings of ***c* -VIII** b, ***t* -VIII** b, ***c* -IX** and ***t* -IX** are in envelope conformation and 6-membered *spiro* -rings of ***c* -VIII** c and ***t* -VIII** c are in the chair conformation [36,39].

For fully heterocyclic amine substituted phosphazenes (cycle E), the Δ(P–N) and δ P_*spiro*_ values of cyclotriphosphazenes having the 7-membered *spiro* -ring ( **I** d and **V** c) are similar, regardless of whether the compounds are mono (type **I** ) and bis (type **V** ) ferrocenyl phosphazenes.

It can be seen from Figure 1i that there are greater changes in Δ(P–N) values for types **II** and **VI** with 1 heterocyclic amine substituent per P atom, types **III** and **VII** with 2 heterocyclic amine substituents per P atom and types **I** and **V** with 4 heterocyclic amine substituents. Therefore, the Δ(P–N) values of these types phosphazenes can be compared with each other according to the number of heterocyclic amine substituents. As expected, the Δ(P–N) value of mono substituted compounds is between the Δ(P–N) value of partly (cycle A) and fully (cycle E) substituted phosphazenes, while geminal substituted derivatives except for **VII** a (cycle D) have the Δ(P–N) value between those of mono (cycle C) and fully (cycle E) substituted ones. The Δ(P–N) value of **VII** a appears to the left more than other geminal substituted derivatives ( **III** a- **III** c) (cycle D) or is greater than those of the fully substituted derivatives (cycle E). This situation may be related to the higher basicity of the DASD substituent in **VII** a. A similar relationship was observed between the Δ(P–N) values of nongeminal *cis* - ( **IV** a) and fully ( **V** d) Van substituted cyclophosphazenes and partly substituted **I** a and **V** a, respectively (Figure 1ii). Furthermore, the Δ(P–N) values of fully heterocyclic amine substituted types **X** (cycle F) and **XI** cyclotetraphosphazenes and types **I** and **V** cyclotriphosphazenes, respectively, are quite close together.

Although the compounds **III** a and **VII** a both have geminal structure and 7-membered *spiro* -ring, and are close in δ P_*spiro*_ shifts, the major difference in their Δ(P–N) values and basicities is that the phosphazenes contain mono and bis ferrocenyl pendant arms, respectively. On the other hand, based on the electron-releasing capacity of the ferrocenyl pendant group for partly substituted phosphazenes (cycles A and B), it has been made the following order: Type **VIII** >type **V** >type **I** . Type **I** ( **Ia** ), and type **V** compounds ( **Va** and **Vb** ) are mono- *spiro* mono and bis structures, while type **VIII** ( ***t* -VIIIa** , ***c/t* -VIIIb** , and ***c/t* -VIIIc** ) phosphazenes are di- *spiro* bis structures. As expected, the electron releasing powers of 2 ferrocenyl pendant groups are greater than those of 1 ferrocenyl pendant group. Moreover, in partly substituted phosphazenes (cycle A), the δ P_*spiro*_ shifts of 7-membered **Ia** and **Vb** are close to each other while 6-membered Va has a lower δ P_*spiro*_ shift.

In the case of 5-membered *spiro* -ring geminal ( **IIIb** and **IIIc** ) and 6-membered *spiro* -ring fully ( **Ib** and **Ic** ) substituted phosphazenes, the electron releasing capacity of DASD group is much larger than that of Pip and Pyr, respectively.

Besides, when the number of atoms increases in the *spiro* -ring, the electron releasing capacity of the phosphazene decreases. In general, the electron releasing power of the rings is in the following order: *spiro* -rings with 5-membered > *spiro* -rings with 6-membered > *spiro* -rings with 7-membered.

As a result, electron−withdrawing substituents, like chlorine group, increase Δ(P–N) values, pulling away electrons from *spiro* -ring/rings to the phosphorus atom bonded to the electron−withdrawing groups. Whereas the electron-releasing substituents, like heterocyclic amines, decrease Δ(P–N) values, resulting in decreased the bond lengths a and a′ and increased the bond lengths b and b′ when compared bond lengths of partly substituted derivatives. Hence, the shortening of the endocyclic P–N bonds and decreased electron charge density at the exocyclic P-N bonds is likely to be a measure of the electron-releasing power of the substituent and the increase in negative hyperconjugation.

The relationship between the Δ(P–N) and δ P_*spiro*_ shifts makes sense in the basicity of the ring nitrogen atoms in phosphazenes. The basicity of the chlorocyclophosphazene ring nitrogen atoms is quite low, and it may be improved by replacing Cl-atoms with electron-releasing substituents on phosphorus. Thus, the basicity of the phosphazene ring nitrogen atoms (N1-PX2 and N2-P_*spiro*_) in fully substituted cyclotriphosphazenes with those in partly substituted ones can be compared. The basicity of N1 atom/atoms in fully substituted phosphazenes appears to have increased due to electron-releasing power of the heterocyclic amine groups, while N2 atom/atoms in partly substituted phosphazenes due to electron-withdrawing power of the chloro groups. As a result, an increase in the electron-releasing power of heterocyclic amine substituents seems to bring about an increase in the basicity of the nitrogen atom (N1) and the negative hyperconjugation.

#### 2.2.2. The correlation of the δ P_*spiro*_ shifts, endocyclic (α), and exocyclic (α′ ) NPN bond angles

**Figure 2:**
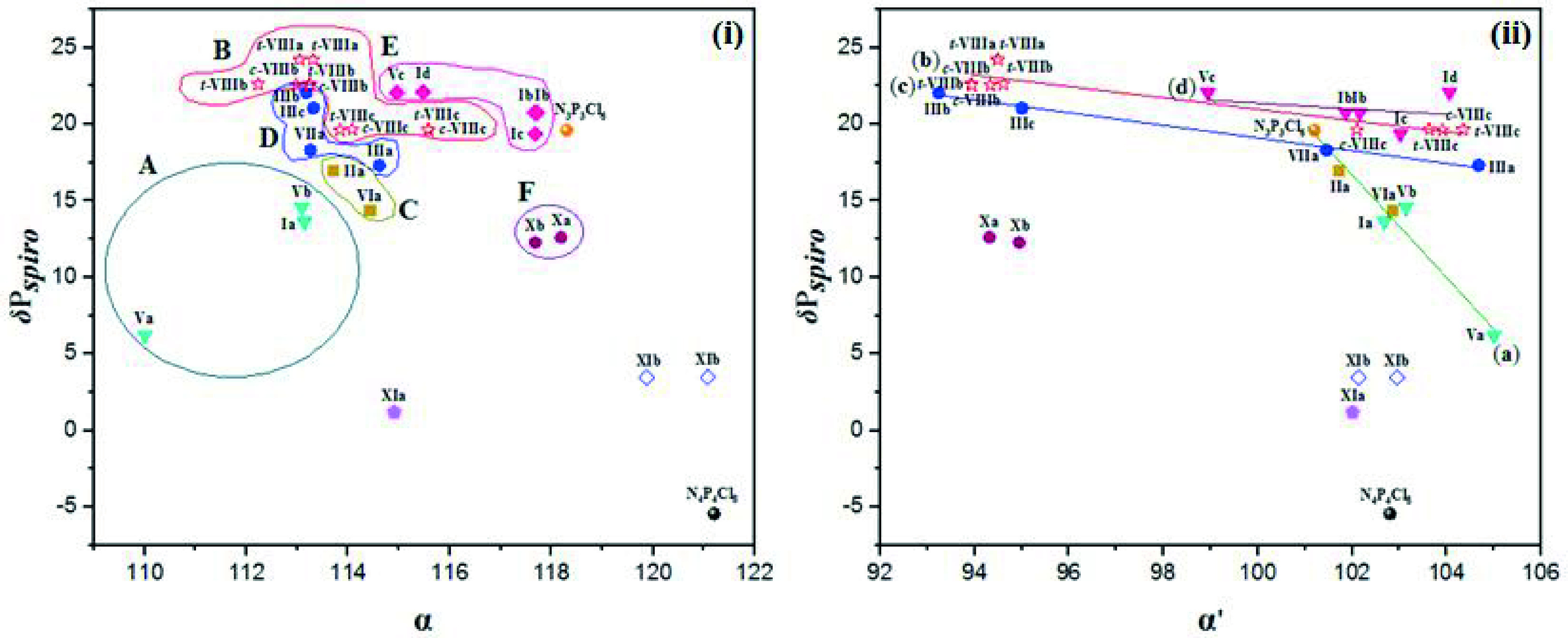
The relationship between δ P_*spiro*_ shifts and endocyclic (α) (i) and exocyclic α ′ (ii) NPN bond angles for partly and heterocyclic amine (Pyr, Pip, Morp, DASD) substituted *spiro* cyclic ferrocenyl phosphazenes. δ P_ClPCl_ shift values of N_3_P_3_Cl_6_ and N_4_P_4_Cl_8_ are 19.60 [56] and –5.45 [58] ppm, respectively. The α and α ′ values are 118.3(2) and 101.2(1)° for N_3_P_3_Cl_6_ [55] and 121.2 and 102.8° for N4 P4 Cl8 [57] respectively.

A cluster of points between the δ P *spiro* shifts and the endocyclic NPN bond angles (α) [A, B, C, D, E, and F given in Figure 2i)] and a trend of approximate linearity between the δ P *spiro* shifts and the exocyclic NPN bond angles (α′) [(a), (b), (c), and (d) given in Figure 2ii] were observed.

**Figure 3:**
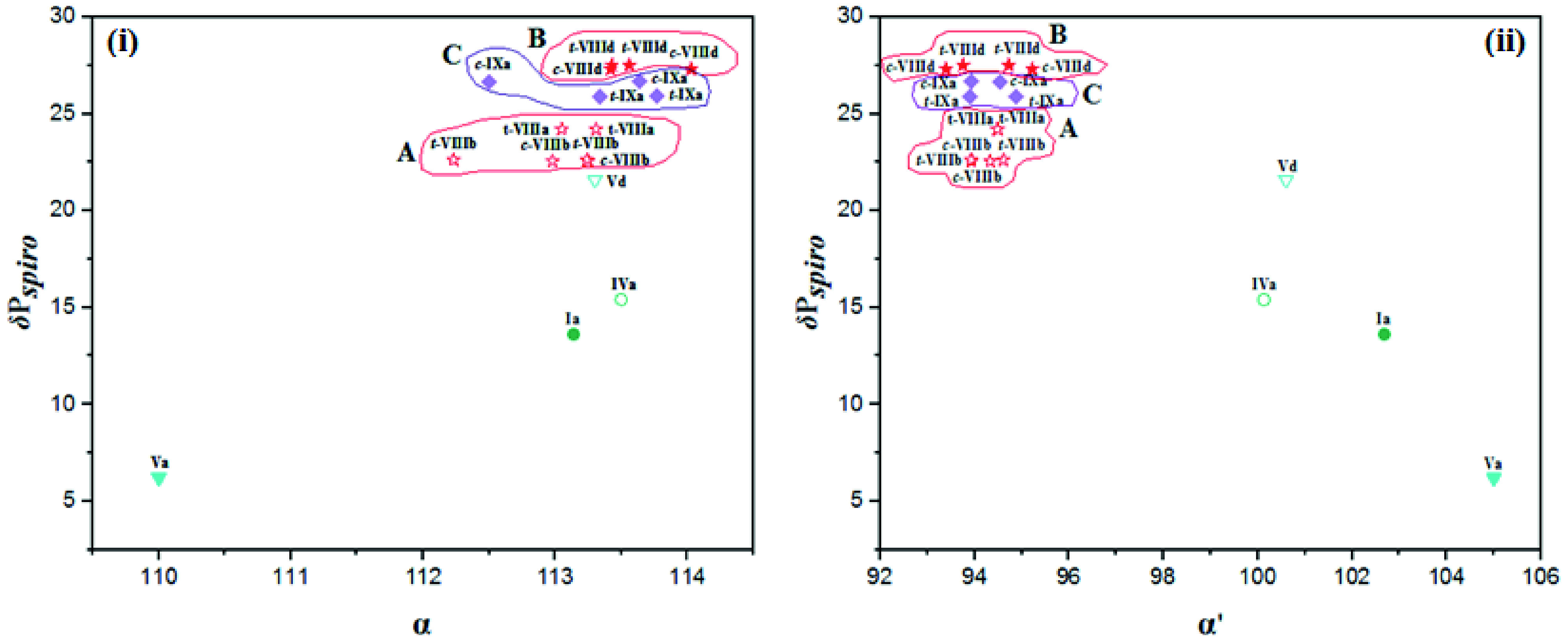
The relationship between δ P_*spiro*_ shifts and endocyclic (α) (i) and exocyclic α′ (ii) NPN bond angles for partly and Van substituted *spiro* cyclic ferrocenyl phosphazenes.

The changes in α and α′ bond angles show parallelism except for a contrasting trend observed for partly substituted types **I** and **V** cyclotriphosphazenes (cycle A) and fully substitute type **X** cyclotetraphosphazenes (cycle F). Small changes in α′ bond angles lead to significant changes in δ P *spiro* shifts. The number of members in the *spiro* -ring seems to be effective on α′ bond angles. In fact, the α′ bond angles of cyclotriphosphazenes with 5-membered *spiro* -ring are narrower than those with larger 6- and 7-membered ones and even narrower than the corresponding angle [101.2(1)°] [55] in the standard compound N_3_P_3_Cl_6_. For example, α′ NPN bond angles of the more flexible 7-membered **IIIa** and **VIIa** are larger than those of 5-membered counterparts **IIIb** and **IIIc** (cycle D given in Figure 2i and line (c) given in Figure 2ii). Likewise, α′ bond angles of 7-( **Vb** ) and 6-( **VIIIc** ) membered phosphazenes are wider with respect to the values of 6-( **Va** ) and 5-( **VIIIa** and **VIIIb** ) membered derivatives, respectively. The α and α′ values of **IIa** are among the α and α′ values of other compounds in cycles A and C (Figure 2i) and line (a) (Figure 2ii) due to its 6-membered *spiro* -ring, and the α′ value of **IIa** close to the α′ angle of the standard N_3_P_3_Cl_6_ [101.2(1)°] [55]. As mentioned before, there is a difference between Δ(P–N) values of the phosphazenes **IIIa** and **VIIa** having geminal structure and 7-membered *spiro* -ring and nearly the same δ P *spiro* shift values. The difference between the α′ and α bond angles of both compounds is ~3 and 1°, and this explains that the α bond angle is less sensitive to the electronic changes. When *spiro* -bisferocenyl Va and VIa cyclotriphosphazenes are compared, it is seen that the δ P *spiro* shift value increases from 6.20 to 14.41 ppm by mono substitution, while the α′ bond angle decreases from 105.0(2) to 102.85(11)° and the α bond angle increases from 110.0(2) to 114.42(13)°, respectively, indicating a change in substituent groups causes a major change in both α and α′ bond angles. In fact, the values of α and α′ bond angles of 7-membered partly substituted cyclotriphosphazene (Vb) are larger and smaller than those of the 7-membered heterocyclic amine substituted cyclotriphosphazene ( **Vc** ). Based on the electron-releasing capacities of the substituents for **Vb** and **vc** , electrons are transferred from heterocyclic amine groups to the cyclotriphosphazene ring in **Vc** and from the cyclotriphosphazene ring to Cl-atoms in **Vb** . The α and α′ bond angles of fully pyrolidine substituted cyclotetraphosphazenes ( **Xa** and **Xb** ) are close to each other, and the angles have the values to be expected for cyclotetraphosphazenes with 5-membered *spiro* -ring. In addition, the α′ angle of the 5-membered DASD substituted **IIIc** is larger than that of the 5-membered Pip substituted **IIIb** , which once again confirms that the DASD substituent has a greater electron-releasing power than the Pip substituent and shows that the electron transferred from the DASD group to the phosphazene ring does not remain only in the phosphazene ring but also transfers towards the *spiro* -ring. In case of partly and fully substituted type **XI** cyclotetraphosphazenes, α angle is much affected by the substitution, but, α′ angle is less affected. Moreover, the correlations between the δ P *spiro* shifts and α (Figure 3i) and α′ (Figure 3ii) NPN bond angles show contrasting trends. For example, the α and α′ angles of 6-( **Va** ) and 4-( **Ia** ) membered partly substituted phosphazenes are smaller than 6-membered fully Van-( **Vd** ) and 4-membered nongeminal *cis-* ( **IVa** ) substituted phosphazenes, respectively.

Although there are few examples of *spiro* -ferrocenyl substituted cyclotetraphosphazenes, the structural parameters of these compounds are given in the figures with the aim of comparison purposes. More values are necessary to learn more about the correlations for cyclotetraphosphazenes.

## 3. Conclusions

A systematic study concerning the correlations between structural parameters [e.g., ^31^P NMR spectral data and X-ray crystallographic data (endocyclic and exocyclic NPN bond angles, and bond lengths)] displayed some characteristic results for mono- and di- *spiro* cyclophosphazene derivatives bearing ferrocenyl pendant arm/arms. Naturally, these results become more reliable when more cyclic phosphazenes from this series are synthesized and the ^31^P NMR spectroscopic and X-ray crystallographic data of these molecules are taken into account. It is necessary to extend the study for other members of the *spiro* cyclic ferrocenyl cyclophosphazene family to get a more general and including views about the correlations between structural parameters of these molecules. Research along these lines is actually under development in our laboratory and results will be presented elsewhere in the forthcoming future.
